# Pentamidine niosomes thwart S100B effects in human colon carcinoma biopsies favouring *wt*p53 rescue

**DOI:** 10.1111/jcmm.14943

**Published:** 2020-02-05

**Authors:** Luisa Seguella, Federica Rinaldi, Carlotta Marianecci, Riccardo Capuano, Mirella Pesce, Giuseppe Annunziata, Fabrizio Casano, Gabrio Bassotti, Angelo Sidoni, Marco Milone, Giovanni Aprea, Giovanni Domenico de Palma, Maria Carafa, Marcella Pesce, Giuseppe Esposito, Giovanni Sarnelli

**Affiliations:** ^1^ Department of Physiology and Pharmacology “Vittorio Erspamer” Sapienza University of Rome Rome Italy; ^2^ Center for Life Nano Science@Sapienza Istituto Italiano di Tecnologia (ITT) Rome Italy; ^3^ Department of Drug Chemistry and Technology Sapienza University of Rome Rome Italy; ^4^ Department of Pharmacy University of Naples “Federico II” Naples Italy; ^5^ Gastroenterology and Hepatology Section Department of Medicine University of Perugia School of Medicine Perugia Italy; ^6^ Pathology Section Department of Experimental Medicine University of Perugia School of Medicine Perugia Italy; ^7^ Department of Clinical Medicine and Surgery University of Naples “Federico II” Naples Italy

**Keywords:** colon cancer, inflammation, pentamidine, S100B‐wtp53, tumour microenvironment

## Abstract

S100B protein bridges chronic mucosal inflammation and colorectal cancer given its ability to activate NF‐kappaB transcription via RAGE signalling and sequestrate pro‐apoptotic *wt*p53. Being an S100B inhibitor, pentamidine antagonizes S100B‐*wt*p53 interaction, restoring wtp53‐mediated pro‐apoptotic control in cancer cells in several types of tumours. The expression of S100B, pro‐inflammatory molecules and wtp53 protein was evaluated in human biopsies deriving from controls, ulcerative colitis and colon cancer patients at baseline (a) and (b) following S100B targeting with niosomal PENtamidine VEhiculation (PENVE), to maximize drug permeabilization in the tissue. Cultured biopsies underwent immunoblot, EMSA, ELISA and biochemical assays for S100B and related pro‐inflammatory/pro‐apoptotic proteins. Exogenous S100B (0.005‐5 μmol/L) alone, or in the presence of PENVE (0.005‐5 μmol/L), was tested in control biopsies while PENVE (5 μmol/L) was evaluated on control, peritumoral, ulcerative colitis and colon cancer biopsies. Our data show that S100B level progressively increases in control, peritumoral, ulcerative colitis and colon cancer enabling a pro‐inflammatory/angiogenic and antiapoptotic environment, featured by iNOS, VEGF and IL‐6 up‐regulation and wtp53 and Bax inhibition. PENVE inhibited S100B activity, reducing its capability to activate RAGE/phosphor‐p38 MAPK/NF‐kappaB and favouring its disengagement with wtp53. PENVE blocks S100B activity and rescues wtp53 expression determining pro‐apoptotic control in colon cancer, suggesting pentamidine as a potential anticancer drug.

## 1 INTRODUCTION

By promoting the release of cytokines, interleukins and other pro‐inflammatory signalling molecules, chronic intestinal inflammation significantly contributes to the carcinogenic microenvironment^1^, ^2^ and genomic instability able to escape the control of tumour suppressor factors, such as *wt*p53.^3^, ^4^ In this context, enteric glial S100B protein overexpression has been linked to the typical features of reactive gliosis, driving the progression from chronic intestinal inflammation to colonic neoplastic lesions. S100B is a neurotrophin, constitutionally and specifically expressed by enteric glial cells (EGCs) in the enteric nervous system, which belongs to a multigene family of diffusible Ca^2+^/Zn^2+^‐binding proteins.^5^, ^6^, ^7^ It is known that S100B protein overexpression correlates with poor prognosis in melanoma^8^, ^9^ and glioma,^10^ and early relapse following curative resection in colorectal carcinoma patients,^11^ suggesting its direct involvement in the perpetuation of a tumour‐promoting microenvironment. At micromolar concentrations, S100B accumulates at the receptor for advanced glycation end products (RAGE) site and such interaction leads to mitogen‐activated protein kinase (MAPK) phosphorylation and nuclear factor‐κB (NF‐κB) activation.^12^, ^13^, ^14^ This event, in turn, promotes the downstream release of pro‐inflammatory cytokines and the transcription of inducible nitric oxide (iNOS). Interestingly, S100B has also been proposed as an inhibitor of *wt*p53,^15^ a key pro‐apoptotic protein linked to colon carcinogenesis. By interacting with the C‐terminus of *wt*p53, S100B prevents its tetramerization and protein kinase C‐mediated phosphorylation, inhibiting the transcriptional and tumour suppressor activity of *wt*p53.^15^, ^16^ The targeting of enteric glial S100B protein and *wt*p53/S100B interaction might thus represent a new strategy in colorectal carcinoma therapy.^13^

In this context, the well‐known anti‐protozoal drug pentamidine^17^ has been shown to disrupt S100B‐*wt*p53 interaction in melanoma and glioma cells, acting as an inhibitor of S100B pro‐cancerogenic activity.^8^, ^18^, ^19^ This evidence has clinically translated into ongoing clinical trials, aiming at testing the efficacy of pentamidine as an anticancer drug, in melanoma patients and in patients with metastatic colon cancer undergoing standard chemotherapy as second‐line and/or third‐line treatment (ClinicalTrials.gov Identifier: NCT00729807 and NCT00809796, respectively). We have previously demonstrated that pentamidine exerts marked anti‐inflammatory effects in a mice model of ulcerative colitis, by likely targeting S100B‐*wt*p53 interaction.^20^ However, the effects and the mechanisms of pentamidine on S100B‐mediated inflammation in colon cancer have not been explored yet.

In the present study, we explored (a) the endogenous expression of glial S100B protein in human colonic mucosa from healthy, peritumoral, ulcerative colitis (UC) and cancer biopsies and its correlation with the expression of pro‐inflammatory markers and pro‐apoptotic factors. We also assessed (b) the effects of increasing concentrations of exogenous S100B protein (0.005‐5 µmol/L) in control biopsies. To maximize pentamidine efficacy to block glial S100B‐induced pro‐inflammatory and pro‐cancerogenic effects, we tested (c) increasing concentrations (0.005‐5 µmol/L) of chitosan‐coated vesicular formulation of pentamidine (PENVE, PENtamidineVEhiculation)^21^, ^22^ in human healthy mucosal biopsies after the challenge with exogenous S100B proteins (5 µmol/L). Finally, we investigated (d) whether pentamidine vehiculation by PENVE (5 µmol/L) was able to rescue mucosal pro‐inflammatory markers and pro‐apoptotic factors in order to evaluate whether S100B/*wt*p53 targeting by PENVE might potentially represent an alternative strategy to the current colon cancer chemotherapy.

## 2 METHODS

### 2.1 Experimental design

We collected surgical specimens of peritumoral and tumoral areas from ten patients diagnosed with colon cancer (6 females; mean age 47 ± 0.5 years). None of the patients had a familial history of colon cancer; hence, all cancers were considered sporadic. Patients were diagnosed with left (5 patients, 2 females) and sigmoid (5 patients, 4 females) sporadic colon cancer with no evidence of lymph nodes or distant metastasis and/or local invasion at pre‐operative staging (T1 or T2, N0, M0). As positive controls, we collected four mucosal biopsies from the recto‐sigmoid region of eight UC patients (5 females; mean age 47 ± 0.7 years) undergoing a colonoscopy for relapse of rectal bleeding. All patients had a proven histological diagnosis of UC with a Mayo 2 score at endoscopy and none of them had dysplastic modifications at routine histopathological examinations. Four recto‐sigmoid biopsies form eight otherwise healthy individuals (2 females; mean age 50 ± 1.1 years) undergoing colonoscopy for colon cancer screening served as controls.

Human colonic mucosal samples were used for the experiments and were divided into four groups, as follows: (a) control group comprising colonic specimens collected from eight controls undergoing colonoscopy for colon cancer screening (6 males; mean age 50 years); (b) peritumoral group comprising surgical specimens of cancer‐free peritumoral areas (distance >5 cm from tumour site) collected from eight patients diagnosed with colon cancer (3 males; mean age 47 years); (c) ulcerative colitis group comprising surgical colonic specimens collected from eight UC patients (3 males; mean age 47 years); (d) colon cancer group comprising surgical specimens of tumoral areas collected from ten patients diagnosed with colon cancer (2 males; mean age 47 years).

**Scheme 1:**
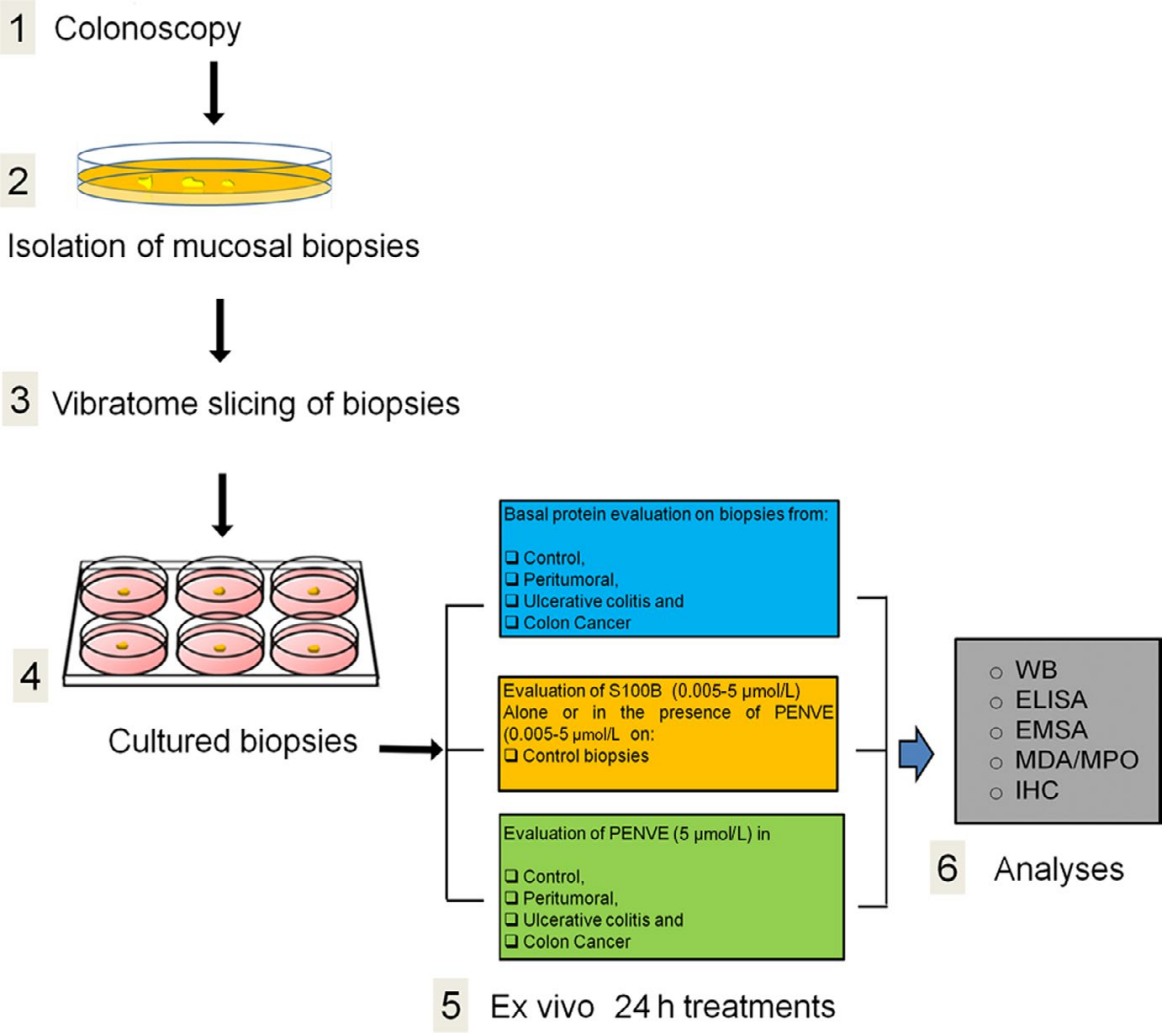
Schematic overview of the experimental design: (1) after colonoscopy and (2) isolation, mucosal biopsies underwent to (3) vibratome slicing and (4) cultured for the scheduled experimental plan. After treatments, (5) the tissues were processed for different analytical procedures

All patients received and signed an informed consent, and all procedures were approved by the ethical committee of the University of Naples ‘Federico II’. Mucosal biopsies were cut in thin slices (400 µm) using a Vibratome VT1200 (Leica Microsystem) to get organotypic culture according to the procedure described.^23^ Specimens were rapidly washed in ice‐cold sterile PBS 1×, orientated and immobilized using cyanoacrylate glue. The vibration amplitude was set at 2.95‐3.00 mm. The slices were then cultured in 6‐well plates in FBS‐supplemented Dulbecco Modified Eagle's Medium (DMEM) at 37°C in 5% CO_2_/95%. In the first set of experiments, specimens were cultured for 24 hours to assess the basal expression of S100B and other pro‐inflammatory and pro‐apoptotic protein expression (see Scheme 1). In parallel, mucosal biopsies from the control group were challenged with increasing concentrations of exogenous S100B protein (0.005, 0.05, 0.5 and 5 µmol/L) orco‐incubated with S100B (5 µmol/L) and PENVE at increasing concentrations (0.005, 0.05, 0.5 and 5 µmol/L) for 24 hours. After the incubation, supernatants were isolated, and specimens underwent homogenization for biochemical and molecular studies. In other experiments, mucosal biopsies from controls, peritumoral, ulcerative colitis and colon cancer patients were cultured as above described, treated with PENVE (5 µmol/L) for 24 hours and subsequently processed for both biochemical and histological procedures (see Scheme 1).

### 2.2 Preparation and characterization of chitosan‐coated niosome of pentamidine (PENVE)

Niosomes were prepared using thin‐film hydration method.^21^ Tween 20 (7.5 mmol/L), cholesterol (15 mmol/L) and DCP (7.5 mmol/L) were dissolved in an organic solvent mixture (chloroform/methanol 3:1 *v/v*) that was then evaporated using rotary evaporator (VV2000, Heidolph) to form a thin ‘film’. The film was hydrated using 5 mL of pentamidine solution (5 mg/mL), vortexed and sonicated at 60°C and 16% amplitude for 5 minutes using ultrasonic microprobe (Vibra‐Cell VCX‐400, Sonics & Materials). The unilamellar vesicle suspension was purified by gel filtration chromatography using Sephadex G75 with HEPES buffer as eluent. The chitosan (low molecular weight) solution was obtained solubilizing chitosan in acetate buffer (0.2 mol/L, pH 4.4) after overnight stirring. The chitosan‐coated niosomes were obtained by adding 1 mL of chitosan solution to an equal volume of uncoated niosomes. The suspension was stirred for 3 hours in a thermostatic water bath at 10°C. Release profiles of pentamidine from PENVE were evaluated in vitro using cellulose membranes.^24^

### 2.3 Western blot analysis

After the above‐described treatments, mucosal biopsies were homogenized in ice‐cold hypotonic lysis buffer (20 mmol/L HEPES, 100 mmol/L MgCl_2_, 0.4 mol/L NaCl, 0.5 mmol/L phenylmethylsulphonylfluoride, 15 mg/mL soybean trypsin inhibitor, 3 mg/mL pepstatin A, 2 mg/mL leupeptin, 40 mmol/L benzamidine, 1 mmol/L dithiothreitol, 1% Nonidet P40, 20% glycerol) and homogenized at the highest setting for 2‐5 minutes in Polytron PT300 tissue homogenizer. Protein concentration was determined using the BioRad protein assay kit. Extracted proteins were thus mixed with gel loading buffer (50 mmol/L Tris, 10% SDS, 10% glycerol 2‐ mercaptoethanol, 2 mg bromophenol/mL) in a ratio of 1:1, boiled for 5 minutes and centrifuged at 10.000 *g* for 10 minutes. Protein concentration was determined and equivalent amounts (50 μg) of each sample were separated under reducing conditions in 15% SDS‐polyacrylamide minigel. The proteins were transferred onto nitrocellulose membrane according to the manufacturer's instructions (Bio‐Rad Laboratories) using iBlot® 7‐Minute Blotting System by Thermo Fisher (Invitrogen), according to the manufacturer's instructions. Depending upon the experiments, the membranes were then blocked by 1‐hour blocking solution consisting of 5% non‐fat dry milk in 1× Tris‐buffered saline (20 mmol/L Tris, 150 mmol/L NaCl) containing 0.05% Tween (TTBS) and then incubated overnight with either mouse anti‐S100B, mouse anti‐iNOS, anti‐RAGE, anti‐PCNA, anti‐Bax, anti‐wild‐type(*wt*) p53 (all from Abcam), anti‐total p38 MAPK, anti‐phospho p38 MAPK (all Cell Signaling Technology, Euroclone, Pero), anti‐AQP4 and mouse anti‐β‐actin (all Santa Cruz Biotechnology). Membranes were then incubated with the specific secondary antibodies conjugated to horseradish peroxidase (HRP) (Dako). Immune complexes were revealed by WesternBright ECL development system (Advansta). Blots were analysed by scanning densitometry (GS‐700 imaging densitometer; Bio‐Rad). All results are expressed as OD (arbitrary units; mm^2^) and normalized on the expression of the housekeeping protein β‐actin.

### 2.4 Electrophoretic mobility shift assay (EMSA)

The EMSA assay was performed to detect NF‐kappaB activation in nuclear extracts of whole‐mount specimens. Double‐stranded oligonucleotides containing the NF‐kappaB recognition sequence for (5′: AGTTGAGGGGACTTTCCCAGCC) were end‐labelled with ^32^P‐γ‐ATP. Nuclear extracts were incubated for 15 minutes with radiolabelled oligonucleotides (2.5‐5.0 × 10^4^ cpm) in 20 mL reaction buffer containing 2 mg poly dI‐dC, 10 mmol/L Tris‐HCl (pH 7.5), 100 mmol/L NaCl, 1 mmol/L EDTA, 1 mmol/L dl‐dithiothreitol, 1 mg/mL bovine serum albumin and 10% (v/v) glycerol. Nuclear protein‐oligonucleotide complexes were resolved by electrophoresis on a 6% non‐denaturing polyacrylamide gel in 1 Tris Borate EDTA buffer at 150 V for 2 hours at 4°C. The gel was dried and auto‐radiographed with an intensifying screen at −80°C for 20 hours. Subsequently, the relative bands were quantified by densitometric scanning with Versadoc (Bio‐Rad Laboratories) and a computer program (Quantity One Software, Bio‐Rad Laboratories). ^32^P‐γ‐ATP was from Amersham. Poly dI‐dC was from Boehringer‐Mannheim. Oligonucleotide synthesis was performed to our specifications by Tib Molbiol (Boehringer‐Mannheim).

### 2.5 NO quantification

NO was measured as nitrite (NO2‐) accumulation in human biopsies supernatants by a spectrophotometer assay based on the Griess reaction as previously described.^25^

### 2.6 Enzyme‐linked immunosorbent assay for S100B, IL‐6 and VEGF

Enzyme‐linked immunosorbent assay (ELISA) for S100B (Biovendor R&D) IL‐6 and VEGF (all from Thermo Fisher Scientific) was carried out on human biopsies supernatants according to the manufacturer's protocol.

### 2.7 Myeloperoxidase assay

Myeloperoxidase (MPO), a marker of polymorphonuclear leucocyte accumulation and general inflammation occurring in colonic tissues, was determined as previously described.^26^ After removal, human colonic tissues were rinsed with a cold saline solution, opened and deprived of the mucosa using a glass slide. The resulting layer was then homogenized in a solution containing 0.5% hexadecyltrimethylammonium bromide (Sigma‐Aldrich) dissolved in 10 mmol/L potassium phosphate buffer and centrifuged for 30 minutes at 20 000 *g* at 37°C. An aliquot of the supernatant was mixed with a solution of tetramethylbenzidine (1.6 mmol/L; Sigma‐Aldrich) and 0.1 mmol/L hydrogen peroxide (Sigma‐Aldrich). The absorbance was then spectrophotometrically measured at 650 nm. MPO activity was determined as the amount of enzyme degrading 1 mmol/min of peroxide at 37°C and was expressed in milli units per 100 mg of wet tissue weight.

### 2.8 Malondialdehyde (MDA) quantification

Malondialdehyde (MDA) was measured with the thiobarbituric acid colorimetric assay in the tissues.^27^ Briefly, 1 mL 10% (w/v) trichloroacetic acid was added to 450 μL of tissue lysate. After centrifugation, 1.3 mL 0.5% (w/v) thiobarbituric acid was added and the mixture was heated at 80°C for 20 minutes. After cooling, MDA formation was recorded (absorbance 530 nm and absorbance 550 nm) in a Perkin Elmer spectrofluorometer and the results were presented as ng MDA/mL.

### 2.9 Immunohistochemistry

After the treatments, mucosal biopsies were fixed in buffered formalin, embedded in paraffin and cut into 5 μm‐thick serial sections. According to the manufacturer's instructions, after heat‐mediated antigen retrieval, the tissue was formaldehyde fixed and blocked with serum. The tissue was incubated with the primary antibodies anti‐S100B (1:50 v/v) or anti‐*wt*p53 (1:50 v/v), (both from Abcam) for 20 minutes. In supplementary experiments, slices were incubated with anti‐MAC387 (1:100 v/v) (Abcam). After three 5‐minute washes, the secondary antibody was added and the samples were incubated at room temperature for 20 minutes. The streptavidin‐HRP detection system (Chemicon Int.) was added and samples were incubated at room temperature. After three 5‐minute washes, 50 μL of chromogen was added and the reaction terminated after 1 minute in water. Sections were then counterstained with haematoxylin‐eosin at room temperature. Negative controls were performed by omitting the primary antibody. Slides were thus analysed with a microscope (Nikon Eclipse 80i by Nikon Instruments Europe), and images were captured at 20× magnification by a high‐resolution digital camera (Nikon Digital Sight DS‐U1). Results were expressed as positive cells per μm^2^.

### 2.10 Statistical analysis

Results were expressed as mean ± SEM of n experiments. Statistical analysis was performed using parametric one‐way analysis of variance (ANOVA) and multiple comparisons were performed by Bonferroni's post hoc test; *P* values <.05 were considered significant.

## 3 RESULTS

### 3.1 Basal pro‐inflammatory and pro‐apoptotic proteins expression profile from ex vivo cultures of control, peritumoral, ulcerative and cancer human colon biopsies

**Figure 1:**
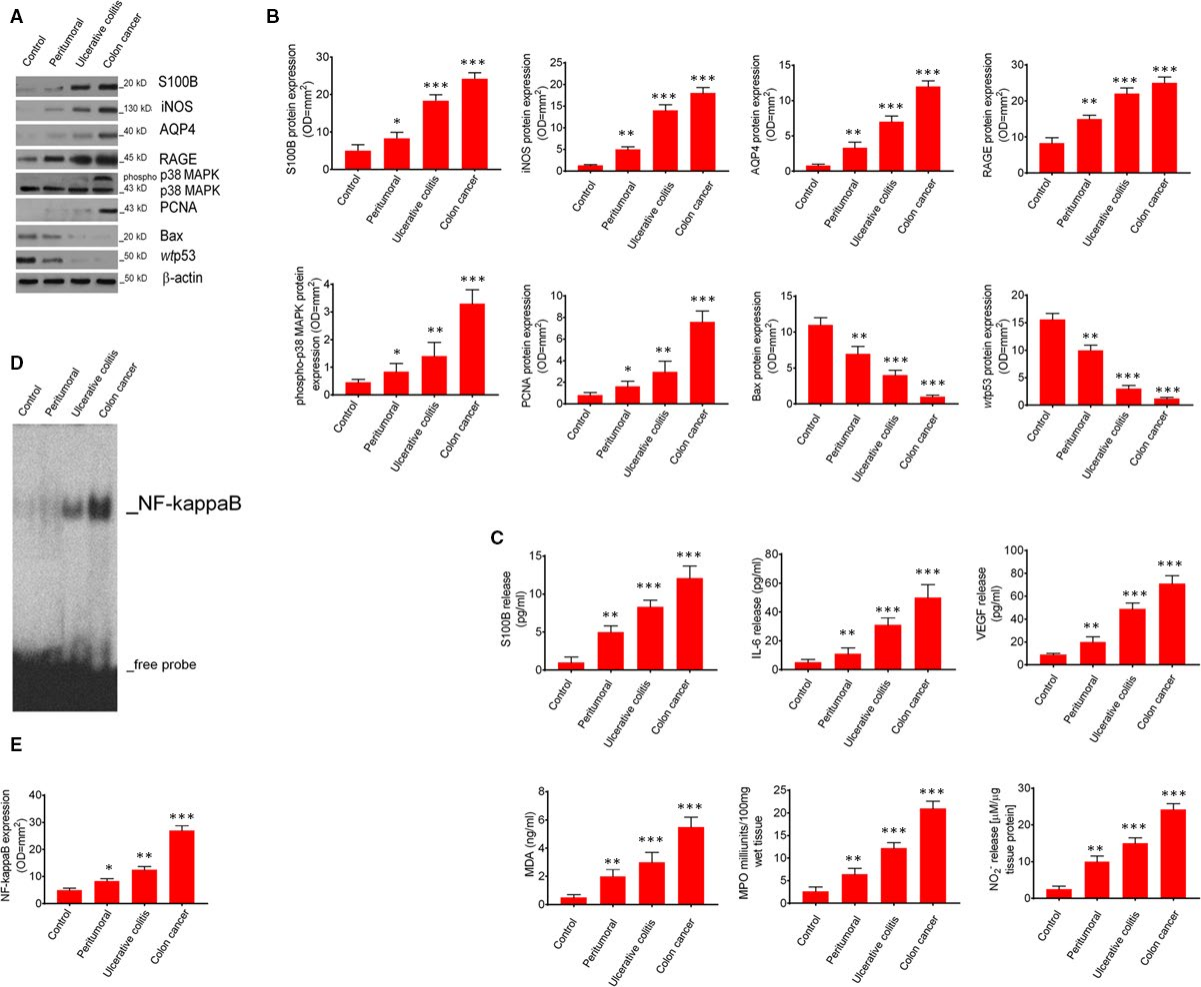
Synoptic framework showing basal correlation among S100B levels, pro‐inflammatory and pro‐apoptotic proteins expression, and signalling molecules release in control, peritumoral, ulcerative colitis and colon cancer biopsies: (A) Immunoblot analysis of the immunoreactive bands showing the progressive increased expression of S100B, iNOS, AQP4, RAGE, phosphor‐p38MAPK and PCNA, and conversely, the progressive decreased expression of Bax and *wt*p53 proteins, respectively, in mucosal biopsies homogenates from control, peritumoral, ulcerative colitis and colon cancer patients; (B) Respective densitometries. (C) This figure also shows the respective quantification of S100B, IL‐6, VEGF, as well as, MDA, MPO and nitrite accumulation, respectively, in control, peritumoral, ulcerative colitis and colon cancer biopsies. (D) EMSA analysis showing the progressively increased expression of NF‐kappaB and (E) its densitometric quantification measured in control, peritumoral, ulcerative colitis and colon cancerpatients' mucosal homogenates. Results are expressed as mean ± SEM n = 5 experiments in triplicate; ***P < .001, **P < .01 and *P < .05, respectively, vs control group

Immunoblot analysis revealed that glial S100B protein expression was sensibly and significantly increased in peritumoral (+67%, *P* < .05), UC (+267%; *P* < .001) and tumour specimens (+384%; *P* < .001) (Figure 1A,B) vs control. In parallel, a significant increase of S100B protein release was observed in peritumoral (+400%; *P* < .01), UC (733%; *P* < .001) and tumour culture medium (1108%; *P* < .001) (Figure 1C) vs control group, respectively. The expression of *wt*p53 and S100B protein was inversely related, yielding to significantly reduced *wt*p53 protein expression in peritumoral (−33.3%; *P* < .01), UC (−82%; *P* < .001) and tumoral specimens (−93%; *P* < .001) (Figure 1A,B) vs control, respectively. Accordingly, there was a marked decrease of pro‐apoptotic Bax protein expression (−39%; *P* < .01; −62.3%; and −91%, both *P* < .001) (Figure 1A,B) vs control.

Conversely, the expression profiles of proliferative proteins displayed an opposite trend in the same biopsy groups, showing a progressive raise in the expression of PCNA (+113%; *P* < .05; +233%; *P* < .01 and +900%; *P* < .001), phospho‐p38MAPK (+50%, *P* < .05; +176%, *P* < .01 +550%; *P* < .001) (Figure 1A,B) and NF‐kappaB expression (+14; +114%; *P* < .05 and +358%; *P* < .001) (Figure 1D,E) in peritumoral, UC and cancer specimens as compared to controls.

VEGF and IL‐6 release and aquaporin 4 (AQP4) expression were assessed as indirect markers of cancer‐related angiogenesis and cell invasion, respectively. Our findings were consistent with a significant progressive increase of VEGF (+122%; *P* < .01 +444%; *P* < .001 and 688%; *P* < .001) and IL‐6 secretion (+127%; *P* < .01 +536%; *P* < .001 and +900%; *P* < .001) (Figure 1C) and AQP4 up‐regulation (+301%; *P* < .01; +653; *P* < .001 and 1306%; *P* < .001) (Figure 1A,B) in peritumoral, UC and tumour specimens, respectively, vs controls. The concentration of myeloperoxidase (MPO) and malonaldehyde (MDA), as well as iNOS expression and mucosal NO production, were used to assess the S100B/RAGE‐dependent induction of oxidative stress and tissue inflammation. We observed a marked and progressive raise in both MPO (+150%; *P* < .01, +372; *P* < .001 and +706%; *P* < .001) and MDA levels (+263%; *P* < .01; +516%; *P* < .001 and +988%; *P* < .001) (Figure 1C) in peritumoral, UC and cancer specimens vs controls. Also, mucosal iNOS protein expression and NO tissue culture medium levels were increased in a similar fashion in peritumoral, UC and tumour samples (+300%; *P* < .01 +1000%; *P* < .001 and 1300%; *P* < .001 vs control) (Figure 1A,B) and (+300%; *P* < .01 +500%; *P* < .001 and +867%; *P* < .001 vs control) (Figure 1C), respectively.

### 3.2 S100B‐induced dysregulation of pro‐inflammatory and pro‐apoptotic protein expression is markedly rescued by PENVE in human mucosal healthy biopsies

**Figure 2:**
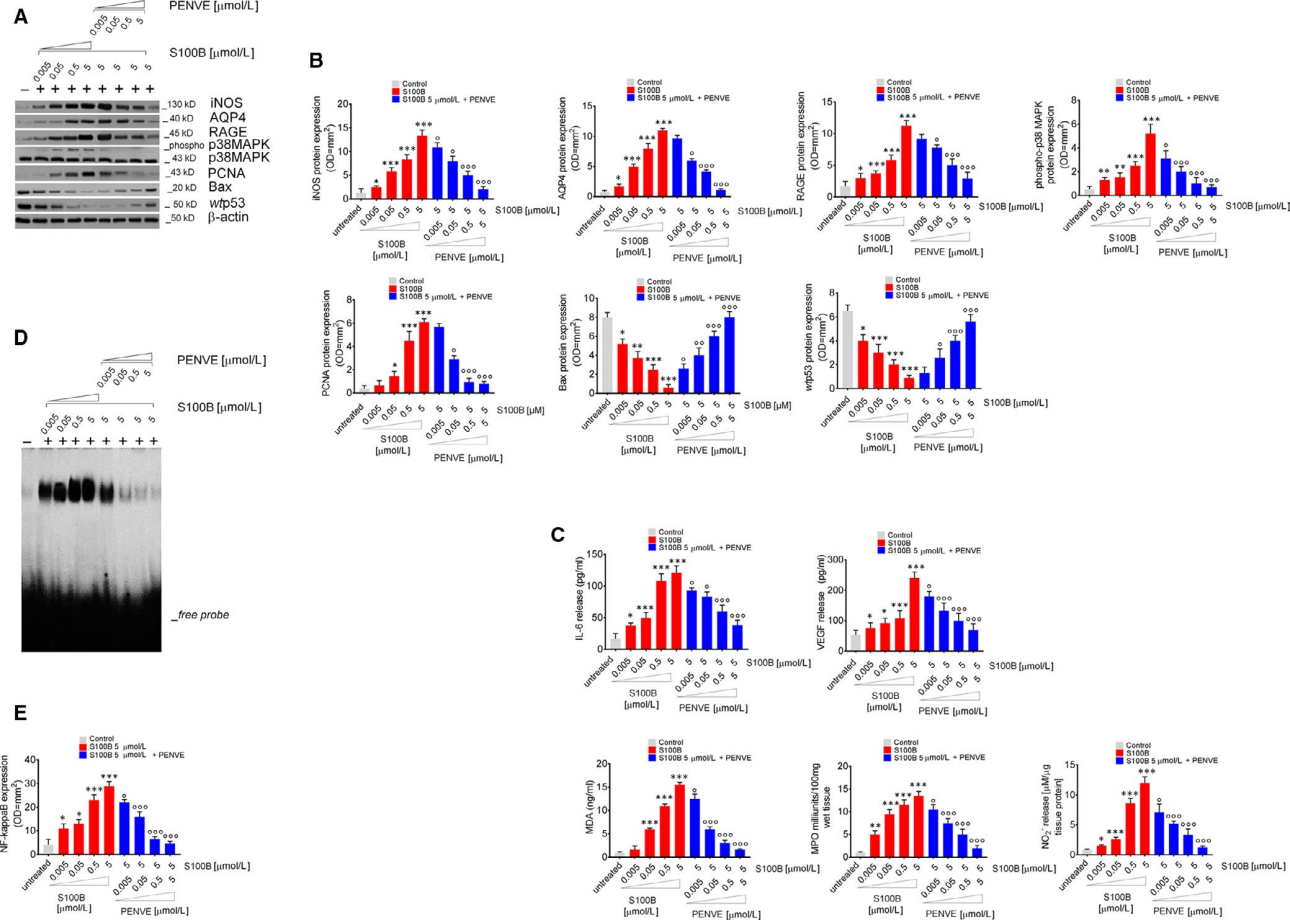
Synoptic framework showing the effect of exogenous increasing concentrations of S100B proteins human control mucosal biopsies and its negative modulation by PENVE. (A) Immunoblot analysis of the immunoreactive bands showing the expression of iNOS, AQP4, RAGE, phospho‐p38MAPK, PCNA, Bax and *wt*p53 in control mucosal biopsies challenged with exogenous S100B protein (0.005‐5 µmol/L) given alone or at the maximum concentration (5 µmol/L) in the presence of PENVE (0.005‐5 µmol/L) at 24 h; (B) relative densitometries. In the same experimental conditions, this figure shows the quantification of (C) IL‐6, VEGF, MDA, MPO and nitrite release from the same treated mucosal biopsies. (D) EMSA analysis showing NF‐kappaB expression and (E) its relative densitometric quantification in the same experimental conditions. Results are expressed as mean ± SEM n = 5 experiments in triplicate; ***P < .001, **P < .01 and *P < .05 vs unstimulated control group and °°°*P* < .001, °°*P* < .01 and °*P* < .05 respectively, vs S100B 5 µmol/L group

To further characterize the role of S100B protein up‐regulation in colon cancer specimens, healthy control mucosal biopsies were exposed to increasing concentrations of exogenous S100B (0.005, 0.05, 0.5 and 5 µmol/L). We found that exogenous S100B induced a marked decrease of *wt*p53 levels in a concentration‐dependent fashion (−38%; *P* < .05; −50%; −69% and −85%, *P* < .001) (Figure 2A,B) vs untreated control biopsies after 24‐hour of incubation. Accordingly, we observed a concentration‐dependent down‐regulation of pro‐apoptotic Bax protein (−31%; *P* < .05; −53%, *P* < .01; −69% and −94% both *P* < .001, respectively, vs control) (Figure 2A,B). Conversely, exogenous S100B increased, in a concentration‐dependent manner, the expression of RAGE (+50%; *P* < .05; +125%; +249% and +574%; *P* < .001 respectively vs control), phospho‐p38MAPK (+100%; +134%, both *P* < .05; +300%; and +934%; *P* < .001), PCNA (+50% n.s; +250%, *P* < .05, +800% and +1300%; *P* < .00) and AQP4 (+101%; *P* < .05; +452%; +874% and +1205%, all *P* < .001) (Figure 2A,B), as well as NF‐kappaB activation (+175%, +225% both *P* < .05; +475% and +645% both *P* < .001) (Figure 2D,E), VEGF (+41% +72%, both *P* < .05; +103%, and +353%, *P* < .001) and IL‐6 release (+125%, *P* < .05; +260%, +550%, and +625%, all *P* < .001) (Figure 2C) vs untreated control biopsies. Moreover, the challenge with exogenous S100B resulted in a concentration‐dependent increase of all the considered markers of oxidative stress, including MDA (+67%, n.s; +567%, +1111% and +1622% all *P* < .001 vs control) and MPO levels (+400%; *P* < .01; +850%, +1050%, and +1250%, all *P* < .001 vs control, respectively) (Figure 2C), iNOS protein expression (+100%, *P* < .05; +366%, +566% and +934%, all *P* < .001 vs control) (Figure 2A,B) and consequent NO release (+51%, *P* < .05; +201%, +904% and +1306%, all *P* < .001 vs control (Figure 2C).

To confirm PENVE ability to counteract the S100B‐related dysregulation of pro‐inflammatory and pro‐apoptotic factors, we evaluated the effects of its increasing concentrations (0.005, 0.05, 0.5 and 5 µmol/L) on whole‐mount control group specimens, after 24‐hour incubation with the highest concentration of exogenous S100B (5 µmol/L). The obtained PENVE is characterized by narrow distribution in dimensions, positive ζ‐potential and drug concentration storage stability in niosomes suitable to obtain an in vitro efficacy.^24^ PENVE treatment led to a significant and concentration‐dependent (0.005‐5 μmol/L) increase of *wt*p53 protein expression (+25%, ns; +125%; *P* < .05, +300% and +450%, both *P* < .001 vs S100B 5 μmol/L) and such effect was accompanied by a significant raise in the expression of pro‐apototic factor Bax (+400%, *P* < .05; +700%, *P* < .01 and +1090% and +1500%, both *P* < .001 vs S100B 5 μmol/L, respectively) (Figure 2A,B) in our experimental conditions. As expected, PENVE administration caused a marked and concentration‐dependent down‐regulation of proliferative markers in cultured mucosal biopsies, reducing the S100B‐induced expression of PCNA (−11%, ns; −50%, *P* < .05; −82% and −86% both *P* < .001 respectively vs S100B 5 μmol/L) and AQP4 (−12% ns; −45%, *P* < .05; −61% and −85% both *P* < .001, respectively, vs S100B 5 μmol/L) (Figure 2A,B), as well as VEGF (−17%, *P* < .05; −45%, −59% and −71% all *P* < .001, respectively, vs S100B 5 μmol/L) and IL‐6 secretion (−17%; −31%, both *P* < .05; −50% and −69%, both *P* < .001 respectively vs S100B 5 μmol/L) (Figure 2C). In parallel, PENVE was able to significantly reduce RAGE expression (−10%, ns; −20%, *P* < .05; −56% and −74%, both *P* < .001, respectively, vs S100B 5 μmol/L) and related activation of p38‐MAPK (−42%, *P* < .05; −61%, −81% and −87% all *P* < .001, respectively, vs S100B 5 μmol/L) (Figure 2A,B) and NF‐kappaB (−19%, *P* < .05; −60%, −80 and −83%, all *P* < .001, vs S100B 5 μmol/L) (Figure 2D,E). PENVE challenge also resulted in a marked inhibition of all analysed markers of oxidative stress, that is MDA (−19%; *P* < .05; −61%; −81% and −90%, all *P* < .001 vs S100B 5 μmol/L) and MPO production (−22%; *P* < .05; −44%; −63% and −85%, all *P* < .001 vs S100B 5 μmol/L) (Figure 2C) iNOS protein expression (−25%, −37%, both *P* < .05; −61% and −84% both *P* < .001, respectively, vs S100B 5 μmol/L) (Figure 2A,B) and NO release (−39%, *P* < .05, −56%, −71 and −89%, all *P* < .001) in the same experimental conditions (Figure 2C). As expected, no‐loaded chitosan‐coated niosome (without pentamidine loading—NpCCN) did not display any detectable effect on S100B‐induced stimuli (data not shown), suggesting that the above‐described effects are specifically related to the release of pentamidine from the niosomes, that is able to disrupt the *wt*p53‐S100B protein‐to‐protein interaction.

### 3.3 Effects of PENVE in ex vivo cultured control, peritumoral, ulcerative colitis and human colon cancer biopsies

**Figure 3:**
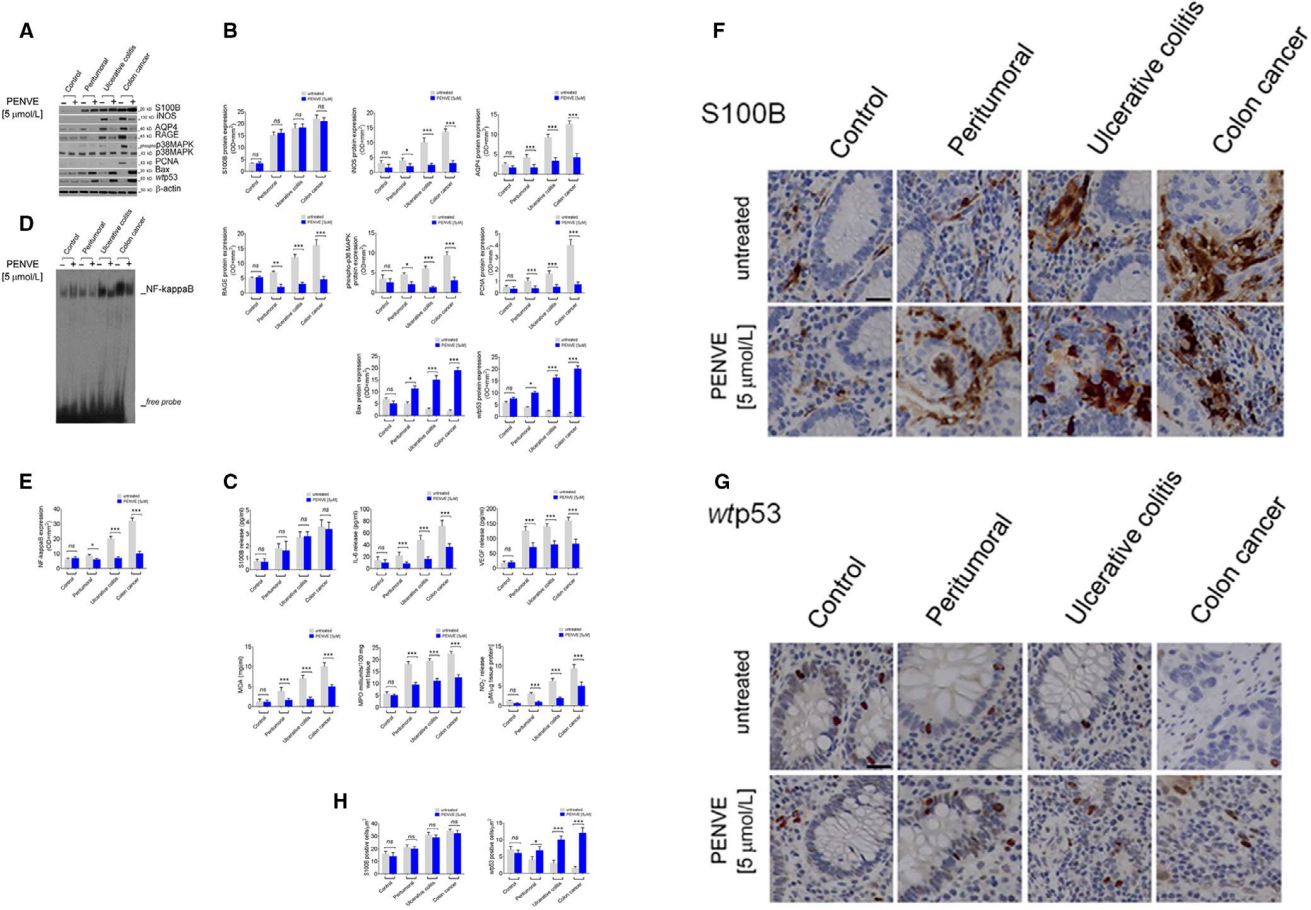
Effect of PENVE challenge in control, peritumoral ulcerative colitis and colon cancer biopsies: (A) Immunoblot analysis of immunoreactive bands referred to S100B, iNOS, AQP4, RAGE, phosphor‐p38MAPK, PCNA, Bax and *wt*p53 protein expression, and (B) their respective densitometries in mucosal homogenates deriving from control, peritumoral, ulcerative colitis and colon cancer cultured biopsies exposed or less to PENVE (5 µmol/L) for 24 h. In the same experimental conditions (C), S100B, IL‐6, VEGF, MDA, MPO and nitrite release in the culture medium of the same experimental group biopsies. (D) EMSA analysis and relative densitometry (E) of NF‐kappaB activation in mucosal homogenates deriving control, peritumoral, ulcerative colitis and colon cancer organotypic cultured biopsies exposed or less to PENVE (5 µmol/L) for 24 h. Photomicrographs showing the inverse correlation between (F) S100B and (G) *wt*p53 expression in immunohistochemical control, peritumoral, ulcerative colitis and colon cancer biopsies, untreated and treated with PENVE (5 µmol/L) for 24 h. (H) Relative graphs showing the relative quantification expressed as positive cells/µm^2^. Magnification 20×; scale bar: 100 μm. For each coupled bar, results are expressed as mean ± SEM N = 5 experiments in triplicate; ***P < .001, **P < .01 and *P < .05 vs respective untreated group

Based on our previous results, we aimed at testing the effects of maximum concentration of PENVE (5 µmol/L) in ex vivo culture of tumoral, peritumoral, UC and control groups' specimens. PENVE did not cause a significant reduction of S100B protein expression and release in the control (−5% and −14%), peritumoral (+9% and +11%), UC (+2% and +3%) and tumour biopsy (+4% and −6%) (Figure 3A‐C) (all *P* not significant) vs untreated control group. On the contrary, the i*n vitro* PENVE challenge induced in all considered experimental groups a significant increase of *wt*p53 protein (+33%, *P* < .05; and +167%, +550 and 1500%, all *P* < .001 vs untreated) with a parallel increased expression of Bax protein (+35%, *P* < .05; and +131%, +500 and +900%, all *P* < .001 vs untreated) (Figure 3A,B). Immunohistochemistry experiments confirmed such effect, showing that S100B levels were not significantly altered by PENVE treatment in all groups (−4%; −8%; 7% and −8% all not significant vs respective untreated group) (Figure 3F,H). Conversely, *wt*p53 expression was inversely correlated with the observed increase in S100B levels and significantly reduced in tumour tissue compared to control tissues (Figure 3G,H). As expected, PENVE treatment was able to restore *wt*p53 expression in our experimental conditions (+46%; *P* < .05; +163%; *P* < .001 and +570% *P* < .001 vs respective untreated group) (Figure 3G,H). Our data also show that a progressive increase of macrophage infiltration was detected in peritumoral (+43%), UC (+101%) and tumour biopsy (+58%), respectively, vs control. In the presence of PENVE, a significant decrease of MAC387 staining in peritumoral (−67%), UC (−62%) and tumour biopsies (−71%) was observed (all *P* < .001 vs untreated) (Figure S1A,B). In line with previous results, PENVE significantly reduced the proliferation and pro‐angiogenic markers release, causing a marked reduction of both PCNA (−10%; n.s; −50%, −75% and −81%, all *P* < .001 vs untreated), AQP4 protein expression (−33%; *P* < .05; and, respectively −60%; −64% and −75%, all *P* < .001 vs untreated) (Figure 3A,B), VEGF (−20%; *P* < .05 and −44%, −43% and −50% all *P* < .001 vs untreated) and IL‐6 release (−18%, n.s; −64%; −71% and 80% all *P* < .001 vs untreated) (Figure 3C) in all the experimental groups vs relative untreated groups. Moreover, PENVE significantly reduced RAGE expression in the peritumoral (−71%, *P* < .01 vs untreated), UC (−75%, *P* < .001) and tumoral group (−76%, *P* < .001 vs untreated) (Figure 3A,B), with a downstream inhibition of oxidative stress, as shown by the decreased MDA levels in the same experimental groups (−18%; n.s; and, respectively −58%; −60% and −77%, all *P* < .001 vs untreated) (Figure 3C). Such effect was thus accompanied by an intracellular decrease of phospho‐p38MAPK (−22%; *P* < .05; and, respectively, −60%; −77% and −89%, all *P* < .001 vs untreated) (Figure 3A,B) and NF‐kappaB activation in all experimental groups (−25%; *P* < .05; and, respectively −45%; −68% and −88%, all *P* < .001 vs untreated) (Figure 3D,E). Finally, a significant reduction of MPO levels (−16%, n.s. and, respectively, −52%; −74% and −81%, all *P* < .001 vs untreated) (Figure 3C), iNOS protein expression(−11%; ns; and, respectively, −26%; −75% and −81%, all *P* < .001 vs untreated) (Figure 3A,B) and relative NO accumulation (−10%; ns; and, respectively −70%; −72% and −80%, all *P* < .001 vs untreated) (Figure 3C) in the tissue supernatants was observed. Analogously, NpCCN challenge did not result in any significant effect (data not shown).

## 4 DISCUSSION

Even though genetic mutations in epithelial stem cells are considered the main step in colon cancer development,^28^, ^29^ compelling recent data indicate that chronic dysfunctions of the surrounding cell microenvironment are critical for cancer development and progression.^30^, ^31^ Among the secreted factors in the pro‐cancerogenic environment, S100B protein is an EGC‐derived neurotrophic protein, commonly up‐regulated in intestinal inflammatory conditions and associated with tumour progression and prognosis.^15^, ^19^, ^32^ Our results have shown a progressive increase in S100B levels human peritumoral, UC and tumour mucosal biopsies, which was correlated with the downstream activation of the proliferative signalling pathway RAGE/MAPK/NF‐kappaB and a parallel reduction of pro‐apoptotic protein wtp53 expression. Besides its effects on cells proliferation and survival factors, the overexpression of S100B protein was also linked to up‐regulation of several well‐known effectors involved in neo‐angiogenesis and invasion of tumour cells, as attested by VEGF and IL‐6 increase and AQP4 up‐regulation, a member of water channel proteins responsible for tumour cells proliferation and invasion.^33^ We also demonstrated that pentamidine is able of inhibiting the S100B‐mediated sequestration of *wt*p53, restoring its ‘genome guardian’ functions in cancer tissue and promoting apoptosis, guarantee prevention of tumorigenic drift through its delivery by PENtamidine loaded VEsicles (PENVE). Non‐tumoral neighbouring cells may promote tumour growth by secreting paracrine signals from the tumour microenvironment.^34^, ^35^ Previous results from our group have demonstrated that S100B protein is a key neurotrophic factor involved in colon inflammation and its targeting by pentamidine is accompanied by significant amelioration of colitis in mice.^20^ Despite chronic inflammatory conditions are well‐established risk factors in colon cancer, there is still limited evidence supporting the role of S100B in the pathophysiology of colon cancer. Our results support that, by fuelling intestinal inflammation, S100B can build a pro‐malignant microenvironment that initiates tumour growth, as supported by the evidence that long‐standing UC is one of the best‐recognized predisposing factors to colon carcinogenesis.^2^, ^36^ In our study, the up‐regulation of S100B orchestrates an increase in oxidative stress and nitric oxide (NO) production, through the increased expression of iNOS. It is well established that lipid peroxidation‐reactive oxygen species (ROS) are among the initiating factors in colon cancer and that pro‐inflammatory cytokines, particularly IL‐6, have been related to tumour progression during chronic inflammatory injury.^37^ In this context, the observation that PENVE significantly reduced the number of tissue‐infiltrating macrophages (Figure S1) suggests that this drug may have a beneficial effect also by reducing inflammatory cytokines from infiltrating immune cells. Interestingly, we have found that the expression profiles of proliferative and pro‐apoptotic proteins, following the exogenous administration of S100B, closely resemble those observed in UC specimens, even in the absence of dysplastic modification of the epithelium. In line with previously reported data, we observed that the function of *wt*p53 and pro‐apoptotic Bax protein were significantly inhibited in UC specimens, further supporting the evidence that the impairment of p53 is an early modification in chronic inflammatory conditions. This evidence is also supported by other studies, showing that in colon cancer patients, an increased immunoreactivity for S100B protein is a reliable prognostic factor of recurrence after curative resection.^38^ S100B‐driven pro‐malignant microenvironment may thus precede cancer development, rather than being its consequence.

**Scheme 2:**
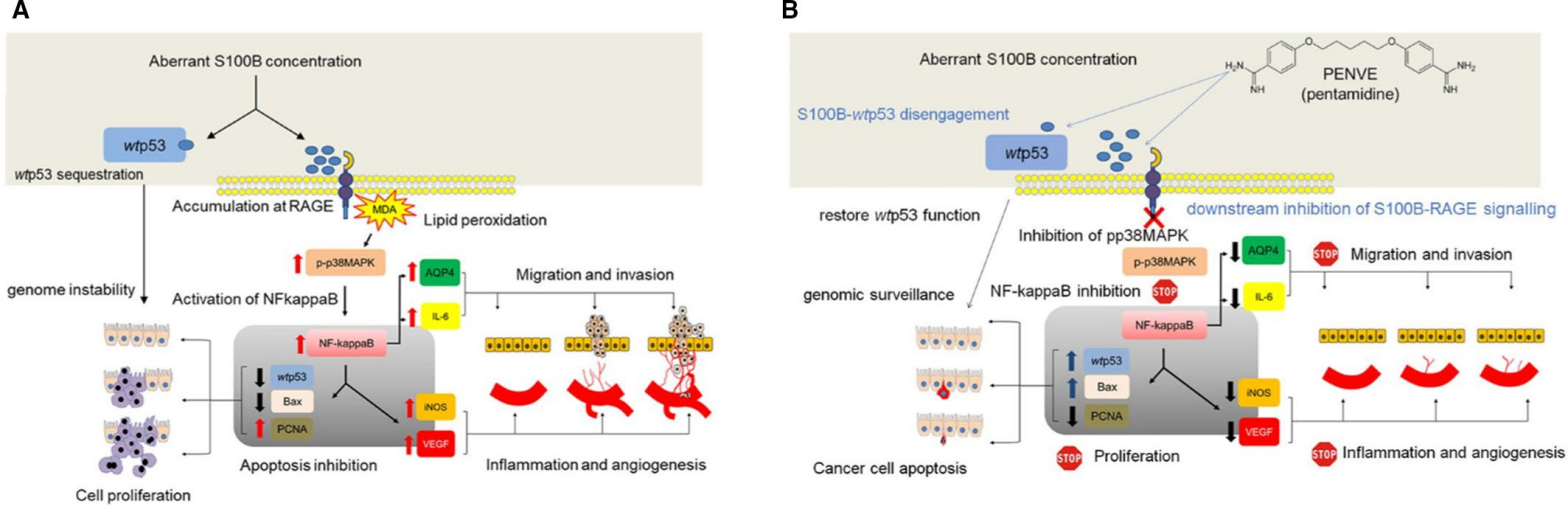
Proposed molecular mechanism leading to carcinogenic drift by S100B aberrant release in tissue milieu and relative pharmacological regulation exerted by PENVE. (A) S100B accumulates at RAGE site and inhibits *wt*p53 functions leading to RAGE‐dependent lipid peroxidation, with consequent phosphor p‐38/NF‐kappaB mediated release of pro‐angiogenic (VEGF), pro‐inflammatory (IL‐6, iNOS) and factors leading to cell migration and invasion (AQP4). Inhibition of *wt*p53 and Bax is accompanied by a massive increase of pro‐proliferative PCNA, with consequent enhancement of cell proliferation and resistance to apoptotic surveillance. (B) In the presence of pentamidine, carried by the PENVE system, the S100B‐*wt*p53 complex is disengaged and wtp53 functions are restored. Consequently, downstream signals due to S100B‐RAGE interaction are down‐regulated, leading to a reduction of VEGF, IL‐6, iNOS, and AQP4 signals. Restored *wt*p53, is accompanied to up‐regulation of Bax and pro‐apoptotic surveillance of scarcely PCNA expressing cell proliferating

As stated before, our major finding was that S100B induced inhibition of *wt*p53 protein was effectively counteracted by the concomitant administration of pentamidine in the PENVE formulation. Pentamidine is a small‐molecule allosteric inhibitor of S100B/*wt*p53 crosstalk, currently tested in phase II clinical trial for malignant melanoma (ClinicalTrials.gov Identifier: NCT00729807). In line with this trend, Oncozyme started a new clinical study aiming at testing pentamidine treatment in patients with metastatic colon cancer undergoing standard chemotherapy as second‐line and/or third‐line treatment (ClinicalTrials.gov Identifier: NCT00809796). However, no results have been published yet and the molecular mechanism(s) responsible for pentamidine anticancer activity remains largely unclear, except for the observation that pentamidine can inhibit oncogenic PRL phosphatases.^39^ This study demonstrates that through the direct inhibition of *wt*p53 function, S100B may contribute to cancer cells' genomic instability, thus increasing the chance to escape to standard anticancer therapies. Although preliminary, our results provide evidence for the first time that pentamidine, by restoring *wt*p53 function in ex vivo culture of colorectal carcinoma specimens, might represent a novel therapeutic strategy in colon cancer. Hence, targeting the tumoral environment and the S100B/*wt*p53 crosstalk might represent an effective complementary strategy in counteracting tumour microenvironment (TME) (Scheme 2).

One of the main limitations to pentamidine use in vivo is its unfavourable colonic bioavailability. Since the drug has been originally developed for the treatment of protozoal lung infections,^17^ it is indeed only available for aerosol or IV injections.^40^, ^41^ The required doses, in order to successfully deliver the compound in colon cancer cells, could, therefore, lead to severe side effects related to its renal and pancreatic toxicity in vivo.^42^ In the PENVE niosomal delivery system, the pentamidine is entrapped into a gastro‐resistant vehicle, easily degraded by bile salts, pancreatic enzymes and the low pH in the gastrointestinal tract. To improve vesicular membrane integrity, niosomal vesicles were coated with polymers including chitosan, pectin and polyethylenglycol (PEG) that act as a bio‐adhesive, biodegradable and hydrophilic polymer, enhancing mucoadhesion with mucosal tissues and promoting interpenetration.^22^ In a future perspective, this improved pharmacokinetic profile could also allow for reducing the orally administered doses in humans, ensuring the best therapeutic results and minimizing the risk of side effects. Although indirectly, this study suggests that enteric glia‐derived S100B promotes colon carcinogenic drift. However, only future investigations will more in‐depth characterize the role played by enteric glia in the pathophysiology of colon cancer. Overcoming the poor colonic bioavailability via this new PENVE formulation, our data also highlight a possible use of pentamidine as a chemotherapy drug, enhancing the efficacy of available tools for colon cancer therapy.

## CONFLICT OF INTEREST

The authors declare that the research was conducted in the absence of any commercial or financial relationships that could be construed as a potential conflict of interest.

## AUTHOR'S CONTRIBUTION

L.S, GS and GE conceived the idea of the present work; FR, CM and MC prepared the niosome encapsulation of pentamidine and verified the analytical methods in vitro. RC, Mi.P and FC performed the immunoblots and other biochemical assays; GA integrated the experimental data and prepared the figures; AS and GB performed the histology and immunohistochemistry experiments; MM and GDP performed the biopsies and collected the human samples; Ma.P., LS, GS and GE wrote the manuscript and supervised the findings of this work. All authors discussed the results and contributed to the final manuscript.

## Data Availability

The data that support the findings of this study are available from the corresponding author upon reasonable request.
