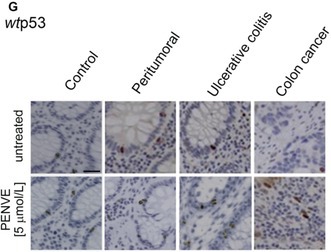# Correction to “Pentamidine Niosomes Thwart S100B Effects in Human Colon Carcinoma Biopsies Favouring wtp53 Rescue”

**DOI:** 10.1111/jcmm.71184

**Published:** 2026-05-12

Seguella, L., F. Rinaldi, C. Marianecci, et al. 2020. “Pentamidine Niosomes Thwart S100B Effects in Human Colon Carcinoma Biopsies Favouring wtp53 Rescue.” *Journal of Cellular and Molecular Medicine* 24, no. 5: 3053–3063. https://doi.org/10.1111/jcmm.14943.

Following concerns raised regarding Figure 3G, the authors have reviewed the figure assembly and identified an issue in the selection of representative image panels, where closely similar microscopic fields were included.

The figure has now been revised by replacing the affected panel with an alternative representative image from the same experimental group and biopsy series. The corrected Figure 3G is provided below.

The authors confirm that this correction does not affect the experimental results, data interpretation, or conclusions of the study. The authors apologize for any confusion caused.

Corrected Figure 3G: