# Supplementary figures and images for: Pentamidine niosomes thwart S100B effects in human colon carcinoma biopsies favouring wtp53 rescue
Source: J Cell Mol Med. 2020 Feb 5;24(5):3053–63. doi: 10.1111/jcmm.14943 (PMC7077541; doi:10.1111/jcmm.14943)

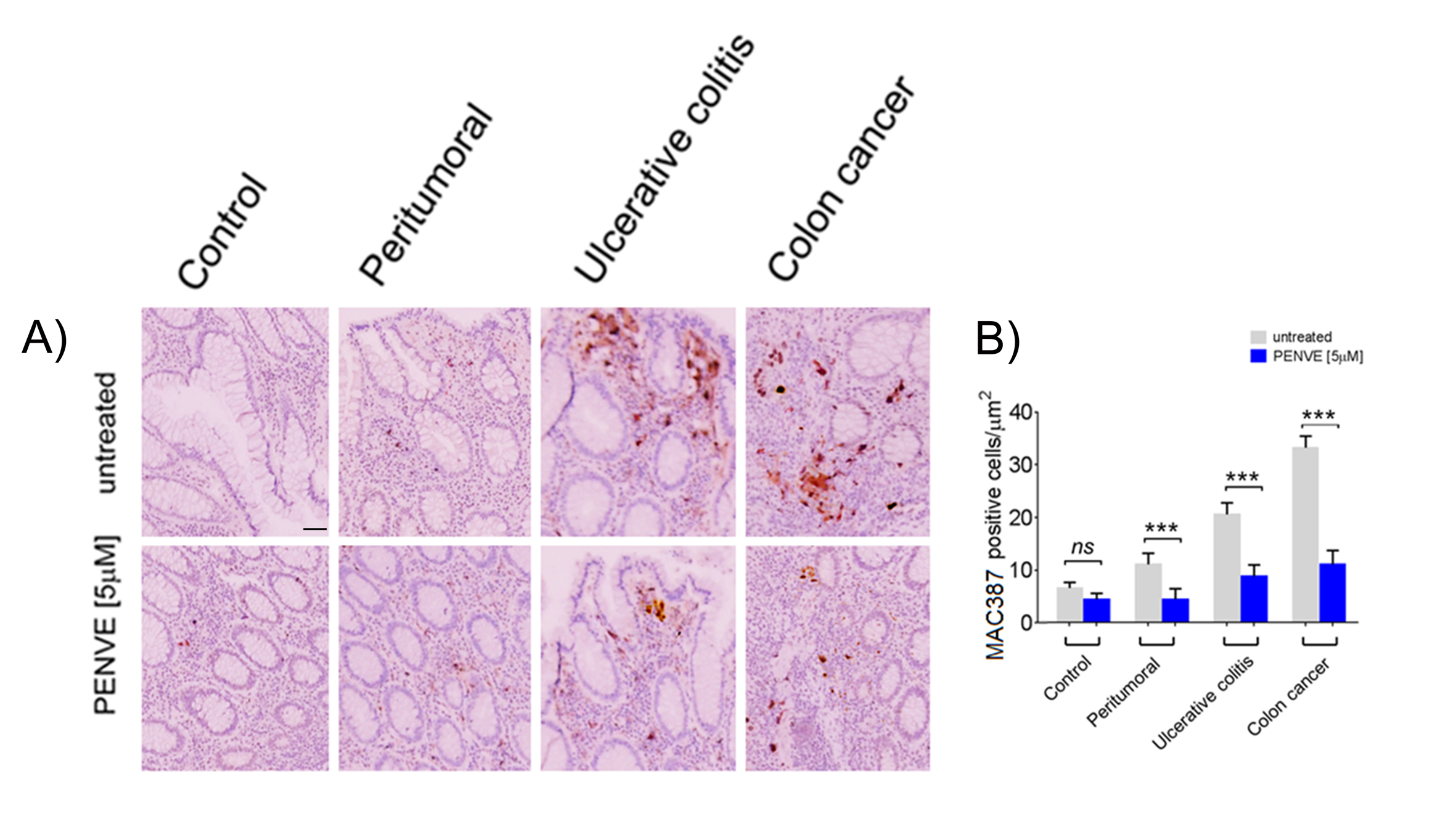

Supplement: Supplementary file 1 [file JCMM-24-3053-s001.jpg]
