# Supplementary material for: Pentamidine niosomes thwart S100B effects in human colon carcinoma biopsies favouring wtp53 rescue
Source: J Cell Mol Med. 2020 Feb 5;24(5):3053–63. doi: 10.1111/jcmm.14943 (PMC7077541; doi:10.1111/jcmm.14943)
Supplement: Supplementary file 2 [file JCMM-24-3053-s002.docx]

Supplementary Figure S1. Effect of PENVE on macrophage infiltration in control, peritumoral, ulcerative colitis and colon cancer: (A) Immunohistochemical images showing MAC387 positive cells infiltration in control, peritumoral, ulcerative colitis and tumoral human biopsies in the absence or presence of PENVE (5 µM). (B) Quantification of the effects of PENVE on MAC387 immunopositive cells in colon criptae; data show the number of MAC387 immunopositive cells per area unit and are expressed as mean±SEM of n=3 experiments. Magnification 10X; scale bar: 100μm. For each coupled bar, results are expressed as mean ± SEM N=3 experiments in triplicate; ***p < 0.001; vs respective untreated group.
